# Trans-eyebrow supraorbital endoscope-assisted keyhole approach to suprasellar meningioma in pediatric patient: case report and literature review

**DOI:** 10.1186/s41016-022-00299-9

**Published:** 2022-09-14

**Authors:** Elizaveta I. Safronova, Suzanna A. Galstyan, Yury V. Kushel

**Affiliations:** 1 Pediatric Neurosurgery Department, Burdenko Neurosurgical Center, 16, 4th Tverskaya-Yamskaya St, Moscow, Russia; 2 Pathology Department, Burdenko Neurosurgical Center, 16, 4th Tverskaya-Yamskaya St, Moscow, Russia

**Keywords:** Pediatric meningioma, Suprasellar tumor, Chiasmal syndrome, Pediatric neurosurgery, Keyhole supraorbital approach, Endoscopic assistance

## Abstract

**Background:**

Meningiomas are rather uncommon tumors in the pediatric population, differing significantly from those found in adults by their atypical location, higher rate of more malignant types, consequently higher risk of recurrence and a less favorable outcome. Even in children, suprasellar meningiomas without dural matrix are rare findings mimicking more common suprasellar lesions.

**Case presentation:**

Here we describe a case of a 12-year-old girl who presented with a rapidly progressing chiasmal syndrome and was diagnosed by MRI with an unusual suprasellar tumor that could not fit the diagnoses expected in a case of a parasellar mass in a child, similar to a craniopharyngioma or optic pathway glioma. After multiple clinical investigations, the tumor etiology was still unclear, so the preferred option of treatment was surgical resection. An endoscope-assisted gross total resection through a supraorbital keyhole approach was performed uneventfully, with total vision recovery in a short time. Benign meningiomas located in the skull base without dural attachment appear to be rare, even in pediatric patients.

**Conclusion:**

Differential diagnoses of suprasellar and para sellar tumor lesions in pediatric patients can be confusing. There are peculiar features of pediatric tumor diseases that should be considered while working out the management strategy. The main principle of meningioma treatment is the highest possible extent of resection minimally affecting the quality of life.

## Background

Meningiomas are known to be the most common intracranial tumors, making up to 15% of all intracranial tumors and over 36% of primary CNS tumors [1, 2]. The World Health Organization (WHO) tumor grading system classifies 15 histologic subtypes of meningiomas into 3 grades correlating to survival, risk of recurrence, outcome and, consequently, required treatment [1, 3]. Patients with grade I meningiomas have the most favorable outcome and they tend to prevail—more than 80% of documented meningiomas. Obviously, the prognosis significantly depends on tumor size, localization, and location-dependent symptoms.

In children under 14 years old, meningiomas are found in less than 2% of cases of brain tumors [4]. In patients in the first two decades of life, meningiomas are diagnosed in up to 5% of brain tumors [5]. WHO grade I makes up 80.5% of all meningiomas in adults and WHO grades II and III make up 17.7% and 1.7% of meningiomas, respectively [1]. The distribution of grades in adolescent meningiomas is less optimistic: up to 54% of grade I with a predominance of meningothelial (41%) and transitional (31%) subtypes; 34% of grade II and 12% of grade III [4, 5]. Thus, meningiomas in pediatric patients are likely to be a rare finding with a higher risk of recurrence and an unfavorable outcome.

In this report, we describe a rare case of a child diagnosed with a benign meningioma arising from arachnoidea of the chiasmatic cistern. In addition, we performed a literature review of meningiomas in children, differential diagnoses of parasellar lesions in children and treatment options.

## Clinical case presentation

**Fig. 1:**
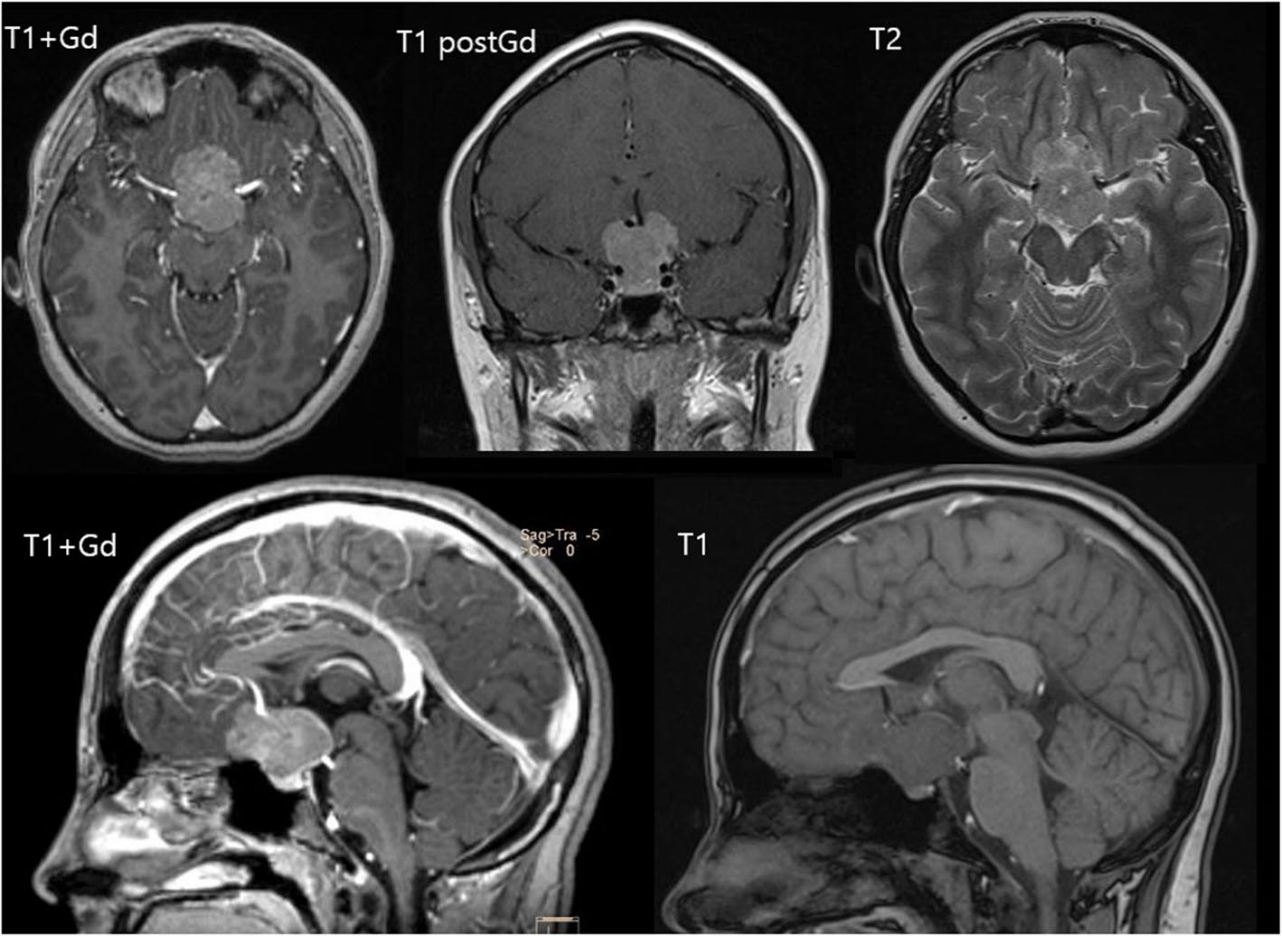
Initial MRI. MRI shows suprasellar tumor that dislocates undamaged 3rd ventricle floor, optic chiasm, and optic nerves rostrally and pituitary gland with its stalk backward. The tumor seems to have no dural attachment or signs of brain invasion

A 12-year-old girl presented to an ophthalmologist complaining of a rapid decrease in her vision. An ophthalmological examination showed low visual acuity (OD = 0.03, OS = 0.09), bitemporal hemianopia, and ophthalmoscopy revealed no pathology. The brain MRI showed a symmetrical suprasellar tumor lesion, evenly intensely enhanced in postcontrast sequences (Fig. 1).

The tumor appears isointense in T1 and T2 and a slightly high signal of tumor mass in the DWI sequence. Unusually for visual pathway glioma cases, the chiasm, both optic nerves and optic tracts are dislocated rostrally. Also notable are intact pituitary gland and infundibular stalk flattened and pushed backwards by the tumor that is unlikely to correlate with pituitary adenoma. Neither any pathological changes in hormone blood tests nor any endocrine symptoms were detected: the results of the investigations of the hormonal profile of the blood at the administration are shown in Table 1 compared with those after surgical treatment and after a 6-month follow-up. Neurofibromatosis of all known types was excluded before administration of the patient to neurosurgeons.

**Table 1:** Hormonal profile of blood serum at the time of first admission, postoperative (day 7) and after 6-month follow-up.

Test name, units	Before surgery	7 days post-op	Follow-up (6 months)	Reference range (13 years old, girls)
**Prolactin,** μmol/mL	229	230	224	110–562
**Tyroid-stimulating hormone (TSH),** mU/L	1.19	0.32	1.08	0.35–4.94
**Triiodothyronine (T3), free,** pmol/L	5.1	3.27	4.3	3.5–6.5
**Thyroxine (T4), free,** pmol/L	14.5	13.7	14.1	9–19
**Parathyroid Hormone,** pmol/L	7.9	8.7	7.95	1.3–6.8
**Cortisol (free),** mcg/dL	16	19	17	5–25
**Follicle-stimilating Hormone (FSH),** IU/L	3.6	3.5	4.2	0.9–8.9
**Lutheinizing hormone (LH),** IU/L	0.9	0.7	1.1	< 0.02–11.7
**Estradiol,** pg/mL	74	71	77	15–85
**Insulin Growth Factor 1 (IGF-1),** ng/mL	187	183	186	90–589
**Growth hormone,** ng/mL	0.88	0.84	0.86	0.01–3.61

In this case, the 12-year-old normally developed girl, having a good general state of health and non-significant medical history, is diagnosed with a suprasellar tumor causing bitemporal hemianopia and reduced visual acuity. Regarding the patient’s age, differential diagnoses include optic pathway glioma, craniopharyngioma, germinoma, pituitary adenoma, intracranial mesenchymal tumor, or meningioma. Optic pathway gliomas are known to cause ophthalmologic symptoms such as eye movement impairment, papilledema, or optic nerve pallor [6] and the patient had none of these symptoms. AFP and b-HCG in the blood are within the normal range. The absence of endocrine disorder and MRI features of the tumor also disprove craniopharyngioma and pituitary adenoma [7]. In addition, it seems impossible to identify the structure tumor originates. All the supposed diagnoses require at least morphological verification, and most of them require gross total resection. The developing visual disturbance forced the parents and neurosurgeons to choose a surgical treatment to remove the tumor and obtain morphological verification.

Having selected a surgical strategy, we considered a minimally invasive trans-eyebrow supraorbital keyhole approach. All basal brain structures including the cranial nerves, third ventricle floor and blood vessels are pushed upwards from the skull base by the tumor’s mass, the visual acuity is lower in the right eye, so the right-sided supraorbital keyhole approach becomes the safest one in this case.

**Fig. 2:**
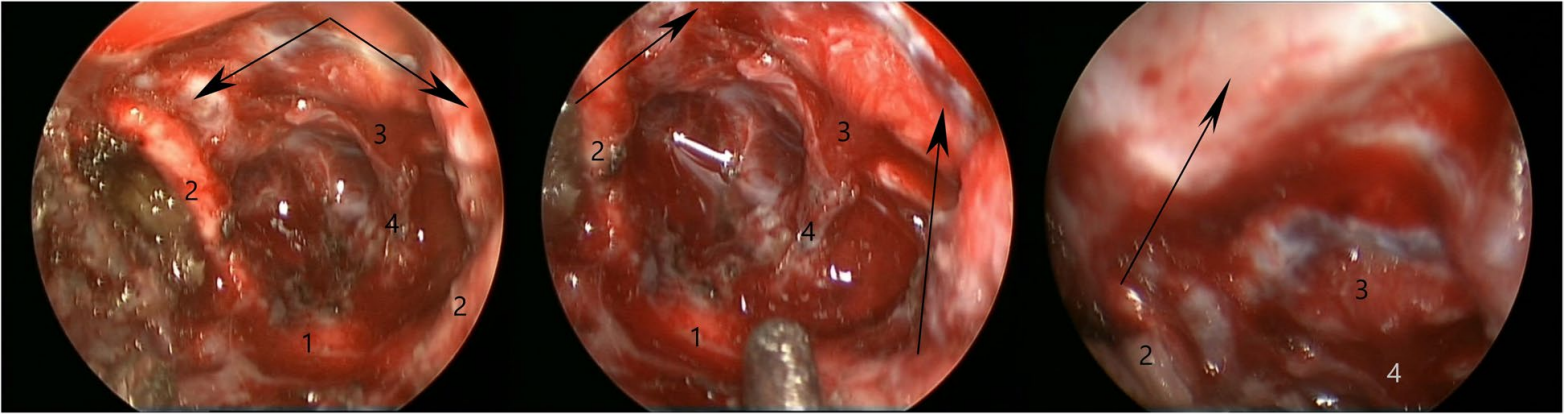
Endoscopic view after tumor resection. These images obtained by a 30°endoscope show dislocated but intact optic chiasm (1) and optic nerves (2), pituitary gland (3) and thin but intact pituitary stalk (4), intact dura (arrows) without signs of meningioma matrix

**Fig. 3:**
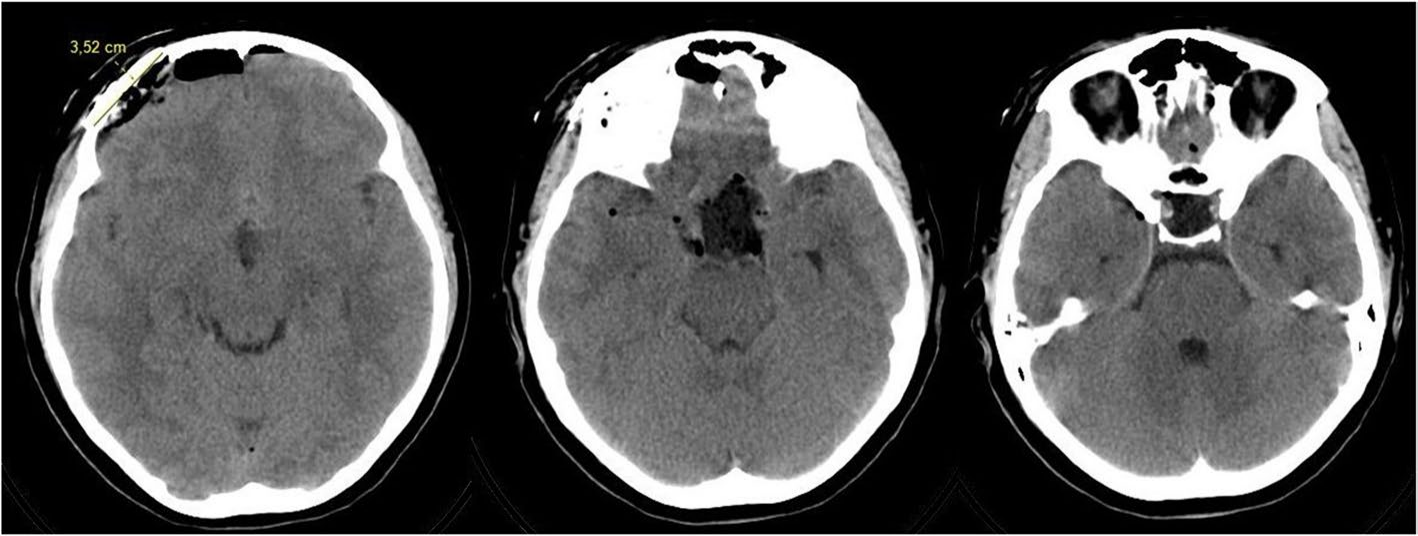
Postoperative (12 h after) CT scan, craniotomy size shown

Intraoperatively, the tumor was found to be totally covered with arachnoidea, leaving the dissection plane between the tumor and the basal brain structures. The tumor was of a dirty-pink color and a soft crumbling texture that appeared to be easily removed piece by piece. The optic nerves, chiasm and pituitary stalk were found intact, as well as the sellar diaphragm and all the underlying dural surface. Endoscopic assistance was used for additional visual control (Fig. 2) to verify that there was no occurrence of hemostasis. The procedure ended uneventfully with the GTR of the tumor. A postoperative CT scan showed no surgical complications (Fig. 3).

**Fig. 4:**
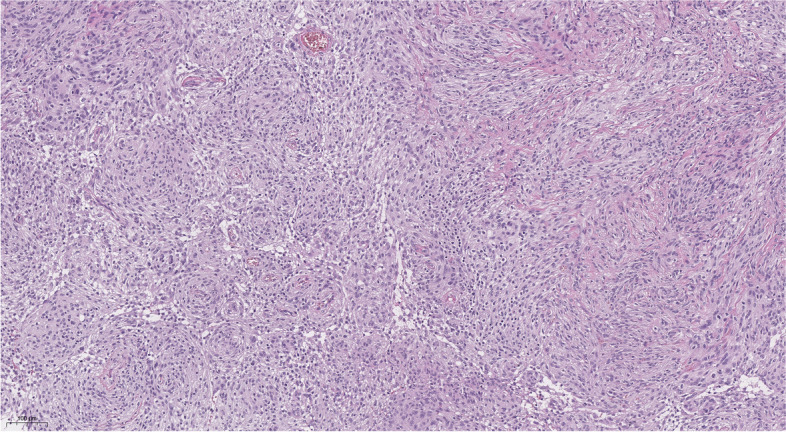
Histological examination, hematoxylin and eosin stain, magnification × 10. Transitional meningioma with whorl (on the left) and fascicules with intercellular collagen (on the right).

The histological examination (Figs. 3 and 4) showed a tumor mostly formed by uniform meningothelial cells. The cells demonstrated a “salt and pepper” nucleus, with a relatively dispersed chromatin pattern and inconspicuous nucleoli. Focally, cells were smaller, with an increased nuclei-cytoplasmatic ratio and uniform nuclei with hyperchromatic chromatin. The cells were arranged in fascicles and whorls with various amounts of intercellular collagen. Focal stromal microcystic degeneration was found. There was a mitotic count of 3 mitoses per 10 high-power fields. The tumor was diagnosed as transitional meningioma, WHO grade I.

**Fig. 5:**
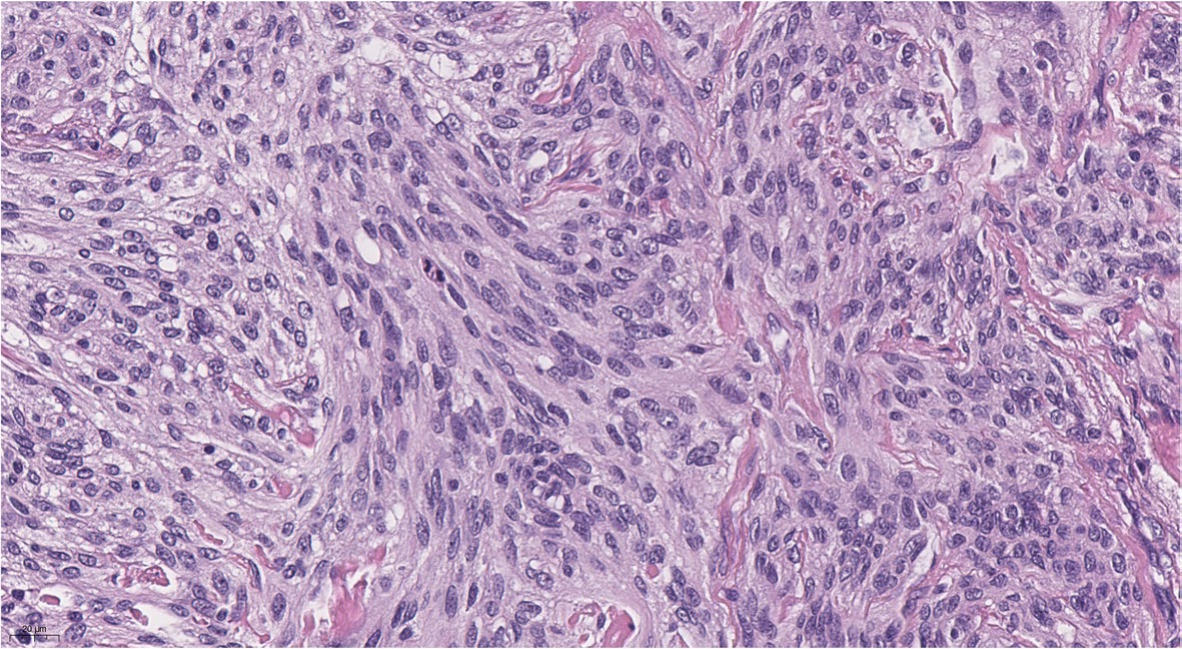
Histological examination, hematoxylin and eosin stain, magnification × 40. This photo captures uniform meningothelial cells and mitotic figures

**Fig. 6:**
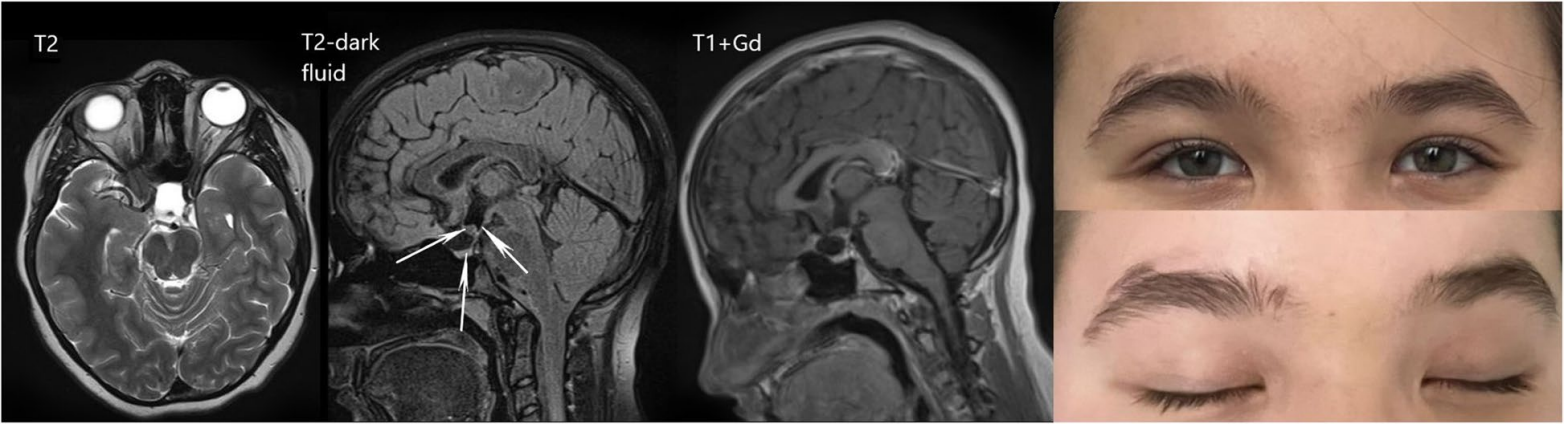
Follow-up in 6 months: MRI and photos of patient. The 3rd ventricle floor, pituitary gland, and optic chiasm are marked by arrows; cosmetic outcome (left eyebrow)

Improvement in the visual function was noticed on the first day of the postoperative period, with increasing visual acuity (OD = 0.1, OS = 0.4) and partial reduction of bitemporal hemianopia. The girl was discharged from the neurosurgical department on the 5th day after surgery with the near-complete recovery of visual acuity (OD = 1.0, OS = 0.8) and persisting moderate bitemporal color vision deficiency. From the first day after surgery, the patient developed diabetes insipidus, conventionally manifesting as polyuria and transitional hypernatremia, and required specific replacement therapy with desmopressin. The dosage of desmopressin was reduced 3 weeks after discharge and after a 1.5-month follow-up, replacement therapy was no longer required. No apparent tumor recurrence or delayed complications were observed during follow-up MRI after 6 months (Figs. 5 and 6). In addition, the patient and her parents mentioned a favorable cosmetic outcome.

## Discussion

Meningiomas originate from meningothelial cells (or arachnoid “cap” cells) that are composed of pia mater, arachnoid mater, tela choroidea, and septae in subarachnoid spaces. Meningothelial cells cover the meninges, organized in a thin layer with tight and gap junctions, providing interaction between neuronal tissue and cerebrospinal fluid and maintaining homeostasis [1, 2]. Meningiomas usually occur in sites of meningothelial cell concentration such as arachnoid granulations, dural sinuses, cranial nerves, and choroidal plexus [8]. It is also remarkable that meningothelial cells of differing anatomic localizations have a different embryologic origin—mesodermal origin in the skull base or neural crest origin in convexity. Histological subtypes of meningiomas and the distribution of somatic mutations in tumors are affected by the features of the cell of origin [9].

The epidemiology of meningiomas in children and adults differs significantly. Making up more than 1/3 of primary CNS tumors in adults, meningiomas are rather unusual for children—less than 2% according to different research. A slight male predominance in pediatric meningiomas with increased morbidity in girls in puberty and the postpuberty stage is related to the significant role of progesterone and estrogen in meningioma pathogenesis [8, 10, 11]. A significant number of cases are associated with neurofibromatosis type 2 and a history of ionizing irradiation [10, 11].

Unusual location is also remarkable in pediatric meningiomas. Adult meningiomas are found mostly in convexity (to 34%), parasagittal (to 22%), and sphenoid or middle cranial fossa (to 25%) [1, 8]. In the pediatric population, the convexity location of meningiomas is still dominant (28.9%), but intraventricular meningiomas are diagnosed in up to 13.6%. Suprasellar and parasellar meningiomas are reported in 1.8–4.4% of pediatric cases [10]. The absence of a dural matrix is a peculiar finding in meningiomas occurring in children—around 24% of all findings, including intraventricular, intraparenchymal, and intra-sylvian localization [10–12]. No cases of suprasellar meningioma without dural attachment were found during the literature review. It is noteworthy, that in large series (in Table 2), authors tend to unite meningiomas of middle cranial fossa regardless the location of dural matrix. The past case series of pediatric intracranial meningiomas are summarized in Table 2, including epidemiological features, tumor location, and histologic grade.

**Table 2:** Largest past case series of meningiomas in children and adolescents

Case series	Study period	***n***	Age	Sex (M:F)	Tumor location (supratentorial only)	NF1	NF2	Histologic grade (%)	GTR, %	Relapse	Mortality
Perilongo et al. 1992 [13]	1975–1991	20	1,5–17	15:5	Convexital–40% (8) Sphenoid wing–10% (2) Parasagittal–10% (2)	1	2	Malignant–45% (9) Benign–55% (11)	60%	8	1
Germano et al. 1994 [14]	1948–1990	23	6–21	14:9	Supratentorial–70% (16) Intraventricular–13% (3)	0	0	Grade I–71% (15) Grade II–29% (6) Grade III–0 Non-specified–9.5% (2)	60%	0	0
Erdinçeret al. 1998 [15]	1968–1994	29	0–15	18:11	Convexital–58.6% (17) Intraventricular–10.3% (3) Intraosseous–3.4% (1) Sphenoid wing–6.9% (2) **Parasellar–6.9% (2)**	7	5	Grade I–93% (27) Grade II–3.4% (1) Grade III–3.4% (1)	86.2%	3	7
Amirjanshidi et al. 2000 [16]	1983–1998	24	4–17	11:13	Convexital–33.3% (8) Parasagittal–12.5% (3) **Parasellar–12.5% (3)** **Suprasellar–4.2% (1)**	0	0	Grade I–95.8% (23) Grade II–0 Grade III–4.2% (1)	75%	6	1
Lund–Johansen et al. 2001 [17]	1972–1998	27	0,3–20	16:11	Convexital–33.3% (9) Parasagittal–14.8% (4) **Clinoid process–11.1% (3)** Sphenoid wing–7.4% (2)	0	3	Grade I–96.3% (26) Grade II–3.7% (1) Grade III–0	92.6%	8	3
Perry et al. 2001 [18]	1972–2000	33	3–18	19:14	Convexital and parasagittal–49% (26) **Skull base–6% (3)** Anterior visual pathway and orbit–8% (4)	1	14	Grade I–40% (21) Grade II–49% (26) Grade III–11% (6)	–	8	5
Rochat et al. 2004 [19]	1935–1984	22	0–14	8:14	Supratentorial: **-Midline–18.2% (4)** -Lateral–68.2% (15)	0	3	Grade I–91% (20) Grade II–9% (2) Grade III–0	68%	9	13
Rushing et al. 2005 [20]	1970–2004	87	0.4–20	52:35	Supratentorial–64% (55) Intraventricular–12% (10)	0	9	Grade I–70.1% (61) Grade II–22.9% (20) Grade III–4.6% (4)	58.6%	15	9
Arivazhagan et al. 2008 [12]	1990–2005	33	5–18	19:14	Convexital–15.2 (5) Intraventricular–24.2% (8) Parasagittal–3% (1) Sphenoidal–6.1% (2) Parasellar/suprasellar–9.1% (3)	3	0	Grade I–75.6% (25) Grade II–9% (3) Grade III–15% (5)	66.7%	6	3
Menon et al. 2009 [21]	1982–2005	38	2.5–20	20:18	Parasagittal–24.4% (10) Skull base–24.4% (10) Convexital–12.2% (5) Intraventricular–7.3% (3)	8	3	Grade I–73.13% (30) Grade II–24.4% (9) Grade III–4.8% (2)	76.3%	7	1
Gao et al. 2009 [11]	1993–2008	54	2.8–18	29:25	Convexital–14.8% (8) Parasagittal–14.8% (8) Sphenoidal ridge–5.6% (3) **Suprasellar–1.9% (1)**	0	5	Grade I–81.5% (44) Grade II–11.1% (6) Grade III–7.4% (4)	72.2%	10	9
Li and Zhao, 2009 [22]	2000–2007	34	2–17	16:18	Convexital–79.4% (27) Parasagittal–5.8% (2) **Tuberculum sellae–2.9% (1)** Sphenoid ringe–2.9% (1) Cavernous sinus–2.9% (1)	0	0	Grade I–79.4% (27) Grade II–8.8% (3) Grade III–11.76% (4)	58.8%	7	6
Lakhdar et al. 2010 [23]	1998–2007	21	2–16	13:8	Convexital–47.6% (10) Parasagittal–23.8% (5) Sphenoidal–9.5% (2) **Suprasellar–9.5% (2)** Intraventricular–9.5% (2)	1	1	Grade I–61.9% (13) Grade II–9.5% (2) Grade III–28.7% (6)	61.9%	7	2
Thuijs et al. 2012 [24]	1974–2010	72	0–18	39:33	Supratentorial–52.7% (38) -Convexital–37.5% (27) Infratentorial–18.1% (13)	10	3	Grade I–73.6% (53) Grade II–18.1% (13) Grade III–8.3% (6)	48.6%	19	7
Ravindranath et al. 2013 [25]	1988–2012	31	0.6–18	22:9	Convexital–42% (13) Skull base–49% (15) Multiple–6.4% (2) Intraventricular–3% (1)	2	2	Grade I–64% (20) Grade II–26% (8) Grade III–9% (3)	83%	20	1
Li et al. 2016 [26]	2005-–2014	44	3–18	20:24	Anterior fossa–16.2% Middle fossa–32.4%: -Sphenoid wing–10.8% **-Parasellar**–**8.1%** -Cavernous sinus–2.7% -Other–10.8%	1	3	Grade I–63.7% (28) Grade II–27.3% (12) Grade III–9% (4)	52.3%	10	4
Grossbach et al. 2017 [27]	1948–2015	39	0–20	15:24	Convexital–58.8% (20) Skull base–55.8% (19)	4	4	Grade I–69% (27) Grade II–26% (10) Grade III–5% (2)	-	15	2
Ilkay et al. 2020 [28]	1994–2001	23	8–18	12:11	Superficial (convexital and parasagittal)–48.2% Skull base (25.9%): -Foramen magnum 8.7% -Retro-orbital 13% -Anterior cranial fossa 8.7% **-Suprasellar/parasellar 0%** Deep (pineal, lateral ventricles, ambient cistern)–11.1%	1	5	Grade I–55.5% (15) Grade II–33.3% (9) Grade III–11.1% (3)	70.4%	10	3
He et al. 2020 [29]	2009–2019	39	1–18	22:17	Convexital–35.6% (14) Skull base–30.7% (12) **-Sellar region–2.5% (1)** Intraventricular–17.9% (7)	2	1	Grade I–66.7% (26) Grade II–25.6% (10) Grade III–7.7% (3)	71.8%	13	2

Case series of pediatric meningiomas (20 and more cases). It takes a long time to obtain data of 20–87 cases because of rarity of meningiomas in children and adolescents. Suprasellar and parasellar meningioma locations are rather rare (in bold) compared to convexital and parasagittal location. It is notable that recurrence rate correlates with presence of neurofibromatosis as well as with the malignancy rate and with the amount of GTR. It is remarkable that most authors do not classify “parasellar” or “suprasellar” meningiomas by the location of dural attachment (clinoid process, tuberculum sellae, etc.). However, this tumor feature can affect the preferred surgical strategy

Known to be mostly (up to 80.5%) benign intracranial tumors in adults, meningiomas are often more malignant in children. Children younger than 3 years old have a worse prognosis in most pediatric tumors due to higher operative morbidity in small children and more probable congenital tumor development, which usually means more aggressive tumor behavior [10]. Children aged 3–12 years old have better overall survival due to the prevalence of WHO grade I meningiomas and less perioperative morbidity and children over 12 years old (in some sources over 14) tend to have tumor features and prognosis very similar to adult patients [5, 10, 11].

Recurrency rate follows the extent of resection, tumor malignancy, association with neurofibromatosis or other family syndromes as are Gorlin syndrome or meningiomatosis. GTR with resection of the dural place of origin or dural attachment is the therapeutic objective both in children and in adults to prevent a recurrence of the tumor. In cases of complicated anatomy of basal tumor or large size, the staged resection might be considered a wise choice [11, 30]. Recommendations on radiotherapy are limited and based on those for adults, so adjuvant radiotherapy may be used to improve outcomes in patients with grade II–III meningiomas, invasion of adjacent brain, or contraindications to surgery. Due to the high risk of radiotherapy in infants, this practice is extremely limited [10, 31].

The choice of a surgical approach to the midline sellar and the suprasellar tumor is based on a principle of surgical control on the structures of deep location, avoiding damage to brain structure and minimizing postoperative morbidity. Initial gross total resection appears to be the most important factor predicting progression-free and overall survival [32]. Endonasal approach to sellar and parasellar meningiomas sometimes makes life easier with early devascularization of the tumor, but in the case of extent, dural attachment possibility of GTR becomes limited [33]. Moreover, this approach is challenging in patients of young age because of small noses, continuing development of air sinuses and possible influence on the growth of facial structures [34]. In the presented case, the endoscopic transsphenoidal approach was contraindicated despite spacious sphenoid sinus and sellar configuration as the pituitary gland lies in the hypophysial fossa and the tumor has a wide contact area with the skull base. In this case, the transsphenoidal approach would require a huge traumatic trepanation of the base of the skull and would lead to injury and destruction of the pituitary gland.

Working out the treatment strategy for a patient of young age with a brain tumor requires consideration of a good functional and cosmetic outcome. In the case of malignant tumors, there is a strong correlation between the patient’s condition at the start of adjuvant therapy that depends on postoperative morbidity and the outcome of complex treatment. And as was mentioned before, the outcome of benign tumor treatment depends mostly on the extent of surgical resection, but regarding the long overall survival of these patients, the functional and cosmetic outcomes of surgery have a major influence on the quality of patients’ further life. In this light, the development of keyhole approaches to intracranial pathology is an essential part of neurosurgical techniques evolution. The keyhole concept is not only about the small craniotomy size but achieving the goal of surgery minimizing the collateral damage [34].

The trans-eyebrow supraorbital keyhole approach becomes a safe alternative for the patient with anterior midline tumors and meningiomas, especially in cases where the parasellar structures are dislocated upwards and there is an alteration of vision [35, 36]. The risk of CSF leakage is absent compared to the transsphenoidal approach and overall postoperative morbidity appears to be less severe. The size of the tumor does not affect the extent of resection while using small craniotomy [37]. There are also some limitations of the supraorbital keyhole approach. The tumor parts extending under the sellar diaphragm or along the middle cranial fossa behind ala minor, especially on the contralateral side, often cannot be visualized properly with a microscope. In the context of individual planning of minimally invasive surgery of skull base meningiomas, the comprehension of relations between tumor and neighboring structures is crucial. A detailed anatomy-based classification of meningiomas of sellar region seems more relevant as keyhole surgery is gaining popularity. In small craniotomy, despite of the high-qualitied preoperative MRI there is still a chance to get confused by the altered anatomy or to miss parts of tumor that are difficult of access. Endoscopic assistance provides high light intensity and high-resolution visualization of deep brain structures that are beyond microscopic view. The main advantage of endoscopy versus the keyhole approach is better visual control over the extent of the tumor resection and hemostasis. To have all advantages of endoscopic assistance in the supraorbital keyhole approach, it is recommended to use an angled endoscope and curved instruments. In transcranial surgery of the skull base meningiomas, endoscopic assistance provides better visualization of the complex surface of the skull base structures increasing the chances of finding and removing the dural matrix, that may remain hidden out of microscopic view. Endoscope facilitates surgical orientation and consequently improves the result of surgery through keyhole approaches [38].

## Conclusion

Pediatric meningioma is an uncommon and sometimes surprising diagnosis given its epidemiologic, histologic, and clinical features. These tumor specifics are to be remembered when making a diagnosis and management strategy for a pediatric patient. The first treatment option for meningiomas is GTR wherever it is possible, minimizing iatrogenic injury and postoperative morbidity. Due to the variability of anatomical and biological features of meningiomas, high rate of familial diseases, and different prognoses every patient requires a tailored approach. In the case of a rare suprasellar location of pediatric meningioma, a trans-eyebrow supraorbital approach amplified with endoscopic assistance appears to be a wise choice providing less collateral without compromising the effectiveness of surgery.

## Data Availability

The data supporting the conclusions of this article are included within the article.

## Abbreviations

MRI: Magnetic resonance imaging
CNS: Central nervous system
WHO: World Health Organization
AFP: Alpha-fetoprotein
b-hGC: Beta-subunit of human chorionic gonadotropin
CT: Computed tomography
CSF: Cerebrospinal fluid
GTR: Gross total resection
NF1, 2: Neurofibromatosis, type 1 and 2
